# EUPopLink Country report - Netherlands

**DOI:** 10.12688/openreseurope.24218.1

**Published:** 2026-07-09

**Authors:** Stijn van Kessel

**Affiliations:** 1 Department of Sociology, Politics and International Relations, Queen Mary University of London, London, England, UK

**Keywords:** Populism, Euroscepticism, Netherlands

## Abstract

This country report, prepared as part of the EUPopLink COST Action, discusses populist Euroscepticism in Dutch party politics, as well as its limited consequences. Criticisms of the EU’s functioning notwithstanding, mainstream parties as well as a large majority of Dutch citizens continue to support membership of the European Union. The salience of European integration as a campaign issue has generally been low. Two far-right parties have – in one case temporarily – taken ‘hard Eurosceptic’ positions, voicing support for a Dutch exit from the EU. Yet the report suggests that populist parties in the Netherlands have been characterised by an ‘instrumental Euroscepticism’ whereby the EU is brought up as a theme insofar as it is seen to help emphasise a political actor’s message and underlying ideological agenda.

## 1. Introduction

This country report, prepared as part of the EUPopLink COST Action ( Andreadis & Vasilopoulou, 2026), and following the EUPopLink Theoretical Framework and guidelines ( Lorimer et al., 2026), discusses populist Euroscepticism in Dutch party politics, as well as its limited consequences. Since the turn of the 21 ^st^ century, Dutch politics has seen the rise and decline of various populist parties, particularly on the socio-cultural right. A few of those have adopted hard-Eurosceptic positions. Both Geert Wilders’s Party for Freedom (PVV) and the more extremist Forum for Democracy (FvD) expressed a desire for the Netherlands to leave the European Union – though the PVV has shifted back to ‘soft Euroscepticism’ more recently.

With such calls these parties did not echo widespread public rejection of EU membership. Historically, the Dutch population generally showed more enthusiasm for EU membership compared with other (founding) members states ( Aarts & van der Kolk, 2006). Eurobarometer data from Spring 2019 suggested that only 8% of the Dutch would vote ‘leave’ in a hypothetical referendum on EU membership (below the EU average of 14%), and that 84% considered Dutch EU membership to be a good thing – only Luxemburgish respondents answered more positively ( European Parliament, 2019: 16; 21). That is not to say that the Dutch have always been overly enthusiastic about the specific course and nature of the EU – the popular rejection by referendum of the Constitutional Treaty in 2005 and the EU-Ukraine association agreement in 2016 are cases in point. Eurobarometer data from Spring 2026 showed that, while still more positive than the EU average, only half of Dutch citizens had a positive image of the EU (versus 21% who had a negative image) ( European Commission, 2026: 16).

This data is congruent with the observation that for many decades there has been a ‘permissive consensus’ among the Dutch population – which generally entrusted political elites with managing the European integration process – although there have also been signs of a ‘constraining dissensus’ at various occasions since the start of the 21 ^st^ century, whereby many citizens rejected the elites’ drive for more integration (see Hooghe & Marks, 2009). Certainly at the time of the Eurozone crisis, the EU also received increased public attention, and the PVV, for a limited time, aimed to explicitly capitalise on disgruntlement with potential Dutch bailout contributions and ‘diktats from Brussels’. The subsequent migrant crisis and Brexit also kept the EU in the news. Yet the salience of European integration in the eyes of the public has generally remained low. In a poll of June 2025, only 5% of respondents mentioned the EU amongst their top three political topics they thought deserved priority ( van der Schelde et al., 2025: 9). ‘Europe’ was close to a ‘non issue’ in the latest parliamentary election campaign of autumn 2025. This suggests that, while recent key events in world politics – not least the war in Ukraine and the second term of U.S. President Trump – have boosted the salience of international politics, they offer no obvious scope for reinvigorated populist Euroscepticism.

## 2. Populist political actors

The most successful populist party family in the 21 ^st^ century – the period this report focuses on – has been the populist radical right (PRR). As the following overview demonstrates, the Dutch PRR is also marked by remarkable fragmentation.

The oldest case that is still represented in parliament, however, has a left-wing socio-economic ideology. The **Socialist Party (SP)** was founded in 1971, but entered Dutch parliament only in 1994, after it departed from staunch communist doctrines and adopted a more populist discourse, which blamed the political establishment for subscribing to neoliberalism and the free-market ideology, thus ignoring the voice of the ordinary (economically marginalised) people. The party reached its electoral high point with 16.6% of the vote in the 2006 national election. While the SP is still habitually identified as a populist party, populism has become a less regular element in the party’s discourse during the past decades ( van Kessel, 2015; Rooduijn et al., 2023, 2024). Meanwhile, its electoral results have deteriorated quite sharply since the mid-2010s; in the October 2025 national election the party received less than 2% of the nationwide vote.

The first right-wing populist party that entered Dutch parliament after the turn of the 21 ^st^ century was the **List Pim Fortuyn (LPF)**, named after its founder who was murdered in May 2002, just over a week before election day. Fortuyn was highly dismissive of the Dutch political elite, especially the incumbent government, and managed to place the issue of immigration and cultural integration of Muslims firmly on the political agenda. Even without its undisputable figurehead, the LPF gained 17% of the vote in 2002, and entered a short-lived and chaotic governing coalition. The electoral appeal of the LPF declined quickly, not least due to continuous infighting, and the party eventually disbanded in 2007.

After the rapid demise of the LPF, Geert Wilders’ **Party for Freedom (PVV)** has established itself as a more radical PRR force in the Netherlands, becoming the dominant PRR party since its entry into parliament in 2006. Wilders blamed political elites for disregarding the concerns and interests of ordinary citizens, and for a raft of social problems, not least related to the spread of multiculturalism and ‘Islamisation’ of Dutch society. Between 2010 and 2012 the PVV provided support for a right-wing minority coalition, in exchange for the implementation of stricter immigration measures. The party reached its high point with 23.5% of the vote in the 2023 national election, becoming the largest party in parliament. The PVV subsequently joined a governing coalition in July 2024 – with Wilders remaining seated in parliament – that lasted less than one year. As in 2012, the PVV leader withdrew support from government after tabling demands that were unacceptable to his coalition partners. With a vote share of 16.7%, the party faced a considerable loss in the early election of October 2025, and the PVV suffered a split in January 2026. Seven MPs broke with Geert Wilders, forming **Group Markuszower**, named after the leading dissident.

Another far-right party represented in Dutch parliament (since 2017) is the **Forum for Democracy (FvD)**. It progressively radicalised its positions, starting to propagate the ‘great replacement theory’ and various other conspiracies about elite control of government and society. The party benefited from the PVV’s recent decline: it more than doubled its vote share in 2025, receiving 4.5% of the vote, and also expanded its nationwide presence considerably after local council elections in March 2026. A more moderate split from the FvD, **Right Answer 21 (JA21)**, similarly benefited from the PVV’s setback, gaining almost 6% the vote.

Finally, the **Farmer–Citizen Movement (BBB)** has also flirted with radical right ideology but can perhaps best be classified as more unusual case of ‘agrarian populism’. It was founded in 2019 as a response to widespread farmers’ protests against nitrogen emission restrictions. It has rhetorically contrasted the cosmopolitan and unresponsive elites with farmers but also ‘ordinary people’ in rural areas more generally, whilst also favouring stricter immigration policy. The BBB performed particularly well in the March 2023 provincial elections (attracting one fifth of the vote), but received less than 5% of the vote in the November 2023 parliamentary election. It then entered the short-lived cabinet in which the PVV also took part, and only received 2.7% of the vote in the October 2025 election.

In terms of international collaboration, those of the above parties that gained seats in European Parliament (EP) have collaborated in transnational Groups. PVV and BBB are represented in the current EP, and are affiliated with the far-right Patriots for Europe (PfE) and the ‘mainstream’ conservative European People’s Party (EPP), respectively. Longstanding PVV leader Wilders has furthermore been a prominent figure in international far-right circles, as illustrated by his appearance at the May 2025 Conservative Political Action Conference (CPAC) in Budapest, Hungary (hosted by his political friend Viktor Orbán). FvD and its leader Thierry Baudet have connections with various alt-right groups and individuals abroad – Baudet for instance appeared in Steve Bannon’s ‘War Room’ podcast. The party has also been alleged to cultivate ties with Putin’s Russia ( Schaart, 2020), and it has continued to call for the lifting of sanctions against the country, and for the normalisation of (trade) relations ( FvD, 2025: 75).

## 3. Positions on ‘Europe’ and the Populism-Euroscepticism nexus

In line with its left-wing populist ideology, the SP has traditionally described the EU as an undemocratic and neoliberal project that served the interests of large companies, and which threatened working conditions and social rights. Yet the party has remained clearly soft-Eurosceptic, not shunning calls for cooperation to tackle cross-border problems and to improve labour rights. The party’s position did not fundamentally change in response to significant political events of the past decade. Brexit was considered by its then leader Emile Roemer to be a ‘wake-up call’, and seen as an opportunity to make Europe less neo-liberal and more democratic; ‘away from the Europe of mutual competition and [towards] building a slimmed-down EU’ ( SP, 2016). The Covid-19 pandemic offered no real scope for increased Eurosecpticism, while the SP in fact saw a role for the EU in increasing diplomatic pressure on Russia and Israel at the time of the Ukraine and Gaza conflicts. Yet it also continued to voice opposition to militarism and the creation of a joint European defence apparatus ( SP, 2024: 21–2). Whilst expressing support for Ukraine, the SP remained sceptical of it accession to the EU, asking for a referendum on any EU enlargement ( SP, 2025: 25). In its 2025 manifesto, the SP continued to call for a social and just (but non-federal and more democratic) EU. The party saw potential in European cooperation as a protective means against the ‘aggressive power politics’ of countries including the U.S. and Russia ( SP, 2025: 24).

Turning to the populists on the right, the short LPF party manifesto notably lacked outspoken Euroscepticm; it declared that the LPF was a ‘proponent of the EU’ as long as national identity and sovereignty – where possible – could be preserved ( LPF, 2002: 7). Whilst still emphasising economic benefits of free trade, the PVV has from the outset described the EU more pejoratively as an undemocratic ‘super state’, and lamented the handing over of sovereignty to ‘Brussels bureaucrats’ (e.g. Wilders, 2005). In the early election campaign of 2012 the PVV radicalised its position on the EU, associating the EU more loudly than before with unwanted immigration, corrupt, undemocratic and incompetent governance, and with large costs for Dutch taxpayers having to prop up insolvent Southern and Eastern European members states in the context of the Eurozone crisis ( PVV, 2012). For the first time, the party proposed to end Dutch membership of the EU, thus moving from ‘soft’ to ‘hard’ Euroscepticism. This position was maintained in the subsequent years and election campaigns, including in the wake of Brexit, even though the PVV increasingly de-emphasised the issue ( van Kessel et al., 2020). In the 2023 national election campaign, the party still supported a binding referendum on Nexit. Its 2025 manifesto spent very little space on ‘Europe’, and did not mention any desire for leaving the EU. The document instead presented the idea to use vetoes to accomplish various opt-outs from EU policies and to ‘overhaul the EU from within’ ( PVV, 2025: 30).

Similar to the PVV, FvD has lamented the loss of sovereignty in various policy areas as a result of EU membership. Yet it has continued to explicitly support a ‘Nexit’ through ‘an intelligent withdrawal’ from the EU, after which the Netherland should join the European Free Trade Association (now comprising Iceland, Liechtenstein, Norway and Switzerland) and re-introduce its own national currency ( FvD, 2025: 24). Re-gaining sovereignty and continuation of economic cooperation have also been key to JA21’s discourse on the EU. But, in line with its positioning as the ‘moderate’ radical right alternative, the 2025 manifesto did not express any desire to leave the EU, instead calling for a ‘much slimmer’ and ‘agile’ EU ( JA21, 2025: 59). BBB has similarly expressed the desire for the EU to return to its core tasks and limiting bureaucracy, while safeguarding the national interest ( BBB, 2025). The party unsurprisingly called for the protection of the (national) agriculture and fisheries industry.

The responses of the right-wing populist parties to recent global political events have been mixed, but in these responses the EU’s role was never a central reference point. PVV and, especially, FvD were vocal in blaming the Dutch government, rather than the EU, for alleged flaws in the approach towards the Covid-19 pandemic (though they did call for closing EU internal borders) ( de Lange, 2022). The EU did not play a prominent role in reactions to the Ukraine War either. Wilders, who had previously hailed Putin as a ‘true Patriot’, has condemned Russia’s invasion of Ukraine, but has mainly framed the war in terms of its consequences for the Dutch people (referring e.g. to inflation and gas prices) ( Nijhuis et al. 2023). FvD has not explicitly supported Russia’s invasion but has laid the blame on Western powers for provoking Putin’s actions. BBB and JA21 have offered stronger (rhetorical) condemnations of Russia and support for Ukraine, but (like PVV and FvD) have not been supportive of Ukrainian EU membership.

Regarding the election of Donald Trump, Wilders has hailed the U.S. president as a ‘brother in arms’ at a conference of the EP Patriots Group ( Klaassen, 2025). Yet he and other PVV politicians also voiced concern that Trump’s policies may not always be aligned with the Dutch interest. BBB leader Van der Plas expressed the need for more EU member state collaboration in the face of the president’s announced trade tariffs ( Business Insider Nederland, 2024). In an April 2005 parliamentary contribution, former FvD leader Thierry Baudet conversely argued that EU membership was detrimental to the Netherlands as it hampered the Netherlands’ freedom to strike a trade deal with the U.S. ( Tweede Kamer, 2025). In its 2025 manifesto, JA21 (2025: 58) distanced itself from ‘rising anti-Americanism’, but did not link trans-Atlantic relations to the issue of European integration. More recent actions of the U.S. president – including threats to invade Greenland and the Iran War – are unlikely to have incentivised Dutch populist right-wing parties to increase their pro-Trump and anti-EU rhetoric.

Generally speaking, the positions of the Dutch PRR towards the EU have not been greatly affected or shaped by international events of the past ten years. These events may in fact have reduced the strategic appeal of a vocal (populist) Eurosceptic discourse, given that they highlighted the costs of leaving the EU (the Brexit process), or fostered the narrative that Europe needs to form a united bloc (versus Russia and Trump). But the PRR’s Euroscepticism remains informed both by populism and nativism: the EU is seen as an undemocratic, unaccountable and elitist organisation that stands far removed from ordinary citizens, and as one that undermines *national* sovereignty or even cultural identity. Similar frames have been adopted by the left-wing SP, even though this party predominantly discussed the EU with reference to socio-economic rather than socio-cultural issues ( Pirro & van Kessel, 2018). Different from the SP, Dutch right-wing populist parties have acknowledged the economic benefits of free trade with other European countries. As will be described next, (occasional) soft-Eurosceptic discourse without an obvious populist frame was primarily the prerogative of traditional mainstream parties.

## 4. Responses to Euroscepticism and consequences

The 21 ^st^ century Dutch political field has been very fragmented, but there are only a few parties that attempted to politicise the issue of European integration (besides the PVV in 2012). The Euro-federalist Volt is the clearest example, and the liberal Democrats’66 (D66) – the surprise winners of the 2025 parliamentary election – have traditionally also supported a strong EU. These are parties that have favoured further European integration, and thus take an adversarial stance towards the fiercest Eurosceptics of the PRR. The three party families that have been dominant in the second half of the 20 ^th^ century – the Christian democrats, conservative liberals and social democrats – have also traditionally supported the central tenets of European integration – albeit less vocally – and viewed EU membership as a cornerstone of economic prosperity, freedom and peace.

The collective support for these traditional party families has dwindled in the past 25 years, whilst particularly the radical right block has grown. As in many other European countries, the result has been that the issue of immigration has become firmly entrenched on the political agenda. Particularly the centre-right parties have adopted an accommodative strategy with regard to this issue, calling for stricter immigration policies ( de Lange, 2018; van Kessel, 2021). But the PRR’s growth has done less to alter the course of mainstream parties on ‘Europe’, both in terms of issue salience – which has remained low in national election campaigns – and concrete positions. In recent years, too, the conservative liberal VVD, social democratic PvdA and Christian democratic CDA remained firmly committed to Dutch EU membership and generally supported European cooperation on cross-border issues to safeguard economic prosperity and security. That is not to say there were no Eurosceptic notes in their discourse. VVD and CDA typically warned against too much supranational interference, and argued the EU should focus on ‘core tasks’. PvdA has called for a more ‘social Europe’, one less based on the principles of market liberalism.

Yet in contrast to the PRR’s *nativism*, its *Euroscepticism* has been fairly inconsequential in terms of influencing political competitors. The same applies to concrete policy impact – and this is perhaps best illustrated by the lack of resistance to joint European measures by the previous Dutch government (2024–2025), in which the PVV took part as the largest coalition party. Research by the investigative journalism platform *Follow the Money* suggests that PVV ministers were hardly active in seeking to block or amend EU policy, while supporting several pieces of supranational legislation in the Council of Ministers ( Teffer, 2025). There was a botched attempt of PVV immigration minister Marjolein Faber to seek a Dutch opt-out from EU migration and asylum policy, but this was not seriously considered – illustrating a more general decline of Dutch influence during the government’s tenure ( Hekster & Ockhuijsen, 2025). Fierce Euroscepticism by the PVV was mainly voiced in national parliament. The party (together with JA21, FvD and BBB) for instance opposed joint European loans to support Ukraine’s military efforts, but this ultimately did not affect the government’s course of action (support). The subsequent government that was installed in February 2026, a minority coalition between D66, VVD, and CDA, has embarked on a more traditional EU-oriented course again.

## 5. Conclusion

Similar to other countries, populist parties in the Netherlands have voiced Eurosceptic rhetoric compatible with their broader ideological nature. The SP has often framed the EU as a neoliberal organisation that furthered the interest of multinationals and the rich, at the expense of (underprivileged) ordinary citizens and workers. Its Euroscepticism has remained ‘soft’, however, and the EU has been portrayed as an organisation that could be reformed into something more benign. The far-right PVV and FvD expressed more radical Euroscepticism, even though the former has recently abandoned its ‘hard’ Eurosceptic position. The fact that the PVV has noticeably changed course (both in terms of position as well as salience), indicates that the EU has not been a stable core issue for the party, even though it has been consistently framed by means of the PRR’s nativist and populist arguments. The issue of immigration has been much more central to the discourse of the PRR, and indeed to the ‘mainstream’ parties which have felt its electoral pressure.

These observations suggest that we can speak of an ‘instrumental Euroscepticism’ whereby the EU is brought up as a theme insofar as it is seen to help emphasise a political actor’s message and underlying ideological agenda. This idea is further reinforced by the observation that, in the Dutch context, the salience of EU membership or European integration has been low: Euroscepticism has been primarily elite-driven, rather than emerging from widespread and active public hostility towards the EU. In the context of Russian aggression and unpredictable foreign policy under the Trump administration – in which calls for more conjoint European action have become louder – it is unlikely that populist actors see much point in further amplifying their hostility towards the EU.

That said, there are clear nuances in terms of the ‘hardness’ of Euroscepticism across the various Dutch populist actors. In a crowded field in which far-right parties have to distinguish themselves from like-minded competitors in order to be electorally successful, ‘Europe’ serves as a potentially useful issue (see Taggart, 1998). Rather than being a topic of key importance as such, the issue of European integration can be used by parties to signal their general position on the ideological spectrum, and their distinctiveness from one another.

## Ethics and consent

Ethical approval and consent were not required.

## Data Availability

No primary dataset is associated with this paper. The study is based on secondary sources, all of which are appropriately cited in the text and listed in the bibliography.
