# Supplementary material for: EUPopLink Country report - Netherlands
Source: Open Res Eur. 2026 Jul 9;6:241. [Version 1] doi: 10.12688/openreseurope.24218.1 (PMC13560592; doi:10.12688/openreseurope.24218.1)
Supplement: Peer review history

# Reviewer report: Reviewer response for version 1

**DOI:** 10.21956/openreseurope.26224.r78635

**Published:** 2026-08-18

**Authors:** Andrei Zhirnov

**Affiliations:** 1 University of Exeter, Exeter, UK

The article seeks to describe and contextualise the views of Dutch populist parties on European integration. It reports that Dutch voters are largely pro-EU, but this issue is not of high salience to them; that Dutch populist right parties are hard Eurosceptics; and that mainstream parties do not accommodate on this dimension. The report is quite detailed and informative; the text is easy to navigate and is well written.

 There are a few steps that could strengthen the article.

1. At the moment, the arguments about voters are supported by 3 isolated data points. Public opinion fluctuates, and a single statistic may not be as informative as a graph or a table that how the statistic changes over time. There are a few recurrent questions in Eurobarometer that have been part of their questionnaires for decades and can be traced over time (e.g., whether the respondent considers the EU a good thing for their country).
2.  Almost all of the report focuses on political parties and does not discuss voters, except in section 1. Section 1 reports that, on average, Dutch voters are quite positive about European integration and that European integration is a low-salience issue for them. I find this discussion quite interesting but somewhat incomplete: it would be useful to close the loop at the end of the report by discussing the implications of voters’ pro-European attitudes for the Euroscepticism (or lack thereof) of the Dutch populist parties.
3. In section 3, the link between PRR’s Euroscepticism and the election of Donald Trump (and, less so, other global events) could be made more obvious. It could be useful to clarify what the reader should expect or conclude from these parties’ responses to these events.
4. There are a couple of places where “yet” is used to indicate a contrast between two statements, but that contrast is not obvious: e.g., in paragraph 3 of section 3, in paragraph 3 of section 4. This might well be the legacy of a previous version of this paper or something that needs further clarification.

Is the case presented with sufficient detail to be useful for teaching or other practitioners?

Yes

Is the work clearly and accurately presented and does it cite the current literature?

Yes

If applicable, is the statistical analysis and its interpretation appropriate?

Not applicable

Are all the source data underlying the results available to ensure full reproducibility?

Yes

Are the conclusions drawn adequately supported by the results?

Yes

Is the background of the case’s history and progression described in sufficient detail?

Yes

Reviewer Expertise:

comparative politics, elections, populism

I confirm that I have read this submission and believe that I have an appropriate level of expertise to confirm that it is of an acceptable scientific standard.

# Reviewer report: Reviewer response for version 1

**DOI:** 10.21956/openreseurope.26224.r77396

**Published:** 2026-08-18

**Authors:** Ben Crum

**Affiliations:** 1 Vrije Universiteit Amsterdam, Amsterdam, North Holland, The Netherlands

This country report offers a solid overview of populist parties in the Netherlands, their stances on European integration, and their impact. As the author is a well-established expert on the topic, the paper is well-informed. It is also rather brief and sometimes almost telegraphic in its writing. But I take that to be intention of this series.

 On the whole I have only relatively minor suggestions that may help to strengthen the paper a bit still.

 I think the paper’s overall conclusion that populist Euroscepticism in the Netherlands is mostly instrumental in nature is apt. However, if that is the conclusion, then the analysis in Section 3 might highlight the two main exceptions to that claim a bit more. The one is the PVV’s attempt with its manifesto for the 2012 national(!) elections to indeed also include the EU as a target of its nativism. This episode is mentioned (on p.4) but could be highlighted more, especially since the party retreated from this strategy later on. The other (which remains more underdeveloped) is the fact that the FvD very much emerged from an outspoken Eurosceptic inclination (more so than the anti-migration sentiments to which the PVV appeals). The FvD has its roots in the No-campaign for the referendum on the EU -Ukraine association treatment and Euroscepticism has also been a primary intellectual driver of its leader Thierry Baudet (as is even reflected by his PhD dissertation). Frontloading these episodes (and the parties’ subsequent reversals on this) a bit more in section 3 would reinforce the eventual conclusion.

 Another nuance is that I would emphasize the FvD’s electoral volatility more in Section 2: it does not just benefit from the PVV’s recent decline (as written on p.4) but had an earlier electoral peak at the 2019 provincial elections, after which it saw a sharp decline.

 One observation that I would add as regards the “Eurosceptic notes” of the established parties (section 4) is the long and broadly shared tradition of a what may be called a soft Euroscepticism on EU finances, pooling of budgets, and other common EU financial facilities. This tradition goes (at least) back to the rather vocal position of VVD Finance Minister Gerrit Zalm in the 1990s and his haggling for a rebate for the Netherlands but was also very pronounced in the Dutch positions towards the EU handling of the eurocrises and the Covid-crisis, and in those years for instance prominently expressed by PvdA finance minister and Eurogroup chair Jeroen Dijsselbloem. In general, Dutch political parties share a strong commitment to enjoy their EU membership on the cheap (and arguably this takes the sting out of any potentially harder Euroscepticism).

 While I completely subscribe to the conclusion about the “instrumental Euroscepticism”, the other point that is suggested remains more elusive to me. I am not convinced by the claim that the particular issue of European integration can be useful for “parties to signal their general position on the ideological spectrum, and their distinctiveness from one another” (cf. the last line of the abstract). If the author wants to stand by this claim, it will help me if he could back it up with a compelling example (even if only a hypothetical one).

 A small question is why the introduction references the Spring 2019 Eurobarometer? At least for the question on EU membership being a good thing (or not), I am pretty sure there must be more recent Eurobarometer results.

 Finally, a few minor phrasing issues;

- Some claims may benefit from explicitly specifying that they apply to the Netherlands rather than being stated in generic terms: “… they offer no obvious scope for reinvigorated populist Euroscepticism [in the Netherlands]” (p.3); “The three party families that have been dominant in the second half of the 20 ^th^ century” – that is, the three-party families that have dominated Dutch politics …
- I don’t see why the second sentence in the second paragraph on p.5 would start with a “Yet”.
- I am not sure about the term “conjoint European action” (p.6); would not “joint European action” suffice?
- In the reference to the recent SP manifesto on “aggressive power politics” (p.4), I would specify that the reference is not just to [any] countries “including” the U.S. and Russia but rather that the manifesto specifically speaks of countries “like” the U.S. and Russia.

Is the case presented with sufficient detail to be useful for teaching or other practitioners?

Yes

Is the work clearly and accurately presented and does it cite the current literature?

Yes

If applicable, is the statistical analysis and its interpretation appropriate?

Not applicable

Are all the source data underlying the results available to ensure full reproducibility?

No source data required

Are the conclusions drawn adequately supported by the results?

Yes

Is the background of the case’s history and progression described in sufficient detail?

Yes

Reviewer Expertise:

EU politics; politics of the Netherlands; populism

I confirm that I have read this submission and believe that I have an appropriate level of expertise to confirm that it is of an acceptable scientific standard.

# Reviewer report: Reviewer response for version 1

**DOI:** 10.21956/openreseurope.26224.r78638

**Published:** 2026-08-17

**Authors:** Márton Bene

**Affiliations:** 1 ELTE Centre for Social Sciences, Budapest, Hungary

The paper provides a thorough and clearly structured overview of the characteristics of Euroscepticism among Dutch populist parties and its broader consequences. It convincingly argues that, for populist parties, Euroscepticism functions primarily as a political communication tool for signalling ideological positions and distinguishing themselves from mainstream parties (“instrumental Euroscepticism”). At the same time, however, Euroscepticism has become increasingly less salient and appears to have few substantive policy implications, while mainstream parties have shown little evidence of adapting their own positions in response. This is most likely related to the demand-side constraints discussed at the beginning of the paper: Dutch society remains relatively supportive of the EU, or at least of maintaining the status quo. While citizens may resist further deepening of European integration, there appears to be similarly little demand for reversing or substantially weakening existing integration. The paper also does an excellent job of demonstrating the nuanced differences in the Euroscepticism of individual populist parties. It is particularly commendable that the analysis considers not only parties’ positions but also the salience they attach to the issue, which adds important nuance to the overall picture. I nevertheless had some reservations regarding two relatively minor points:

- The discussion of citizens’ attitudes towards the EU, including support for leaving the EU, relies primarily on a single data point from 2019, supplemented by a brief reference to data collected in 2026. First, I find the 2019 evidence somewhat dated for assessing contemporary support for EU membership, particularly since attitudes and preferences regarding the EU are presumably measured regularly in public opinion surveys. Second, because the review of party positions covers approximately two and half decades, it would be useful for the demand-side discussion to adopt a similarly longitudinal perspective rather than focusing largely on a single point in time. Even if this is not the paper’s primary focus, some indication of how attitudes towards the EU evolved between 2000 and 2026 would provide valuable context for interpreting changes in party-level Euroscepticism.
- As the first part of the paper notes, there have been several important instances in which Dutch citizens actively opposed further European integration, most notably the 2005 referendum on the Constitutional Treaty and the 2016 referendum on the EU–Ukraine Association Agreement. I suspect that these referendums also represented important moments in the development of Euroscepticism among Dutch populist parties. However, the paper does not discuss the roles these parties played in the respective campaigns, the positions they adopted, or the possible longer-term consequences of these episodes for their Eurosceptic strategies. Addressing these cases, even briefly, could strengthen the historical account of the evolution of populist Euroscepticism.

There is also a minor typographical error on page 5: in the discussion of Thierry Baudet’s parliamentary argument, the date should be 2025 rather than 2005.

 Overall, I congratulate the authors on this informative and well-executed overview.

Is the case presented with sufficient detail to be useful for teaching or other practitioners?

Yes

Is the work clearly and accurately presented and does it cite the current literature?

Yes

If applicable, is the statistical analysis and its interpretation appropriate?

Not applicable

Are all the source data underlying the results available to ensure full reproducibility?

No source data required

Are the conclusions drawn adequately supported by the results?

Yes

Is the background of the case’s history and progression described in sufficient detail?

Yes

Reviewer Expertise:

political communication, political behavior

I confirm that I have read this submission and believe that I have an appropriate level of expertise to confirm that it is of an acceptable scientific standard.

# Reviewer report: Reviewer response for version 1

**DOI:** 10.21956/openreseurope.26224.r77399

**Published:** 2026-07-31

**Authors:** Alexander Alekseev

**Affiliations:** 1 University of Helsinki, Helsinki, Finland

Having read the EUPopLink country report on the Netherlands, I am happy to congratulate its author on the high quality of his work.

 Although the report is clearly bound by strict formatting and length limitations, it still offers the reader a thorough overview of populism, Euroscepticism, and the interactions between the two in the Dutch context.

 The report is clearly structured, well written, and covers all the key areas it intends to cover, which is important when it is used to develop policy recommendations or support students’ learning process.

 Therefore, my comments and recommendations focus on several comparatively underdeveloped areas that warrant attention to introduce greater clarity or enhance the analytical value of the piece.

- The right-left distinction needs to be conceptualised more clearly. In the Introduction, the author mentions that the right is defined in socio-cultural terms while the left is in socio-economic terms. This point needs greater attention as, at present, the left and right are structured along two different dimensions.
- The report focuses on the party system, and the primary political actors here are political parties. While it is fully valid from the standpoint of analysis, the role of individuals, like both party leaders like Geert Wilders and prominent activists like Eva Vlaardingerbroek. The latter play the central role in representing the Netherlands in the transnational illiberal right-wing movement and shaping mass attitudes toward the EU, which is crucial in the context of their active participation in the movement structured through opposition to the EU in its current shape 
  - Maher, H. (2026). The neoliberal Great Replacement theory: On the racialization of markets and migration. *Distinktion: Journal of Social Theory*, *27*(1), 83–101. refer 1
- This observation leads me to another important point. While Euroscepticism as approached in the report seems to amount to outright rejection of the European Union as such, an important gap remains unaddressed if one does not look into the kinds of Europe that the mild Eurosceptics are willing to offer. What do the parties in question say and do to change the EU from within? What is the place of the concept of the Europe of Nations, arguably advanced by the political parties belonging to the ECR, PfE and ESN?
- In this context, the discussion of international stances would strongly benefit from an analysis of political positions on the Israel-Palestine conflict and the place of Europe in this regard: philosemitism has arguably become crucial for the PRR not only to target the Muslim Other in the Netherlands, but also to criticise the European Union and the European project in general on the civilisationist grounds.
  - Chryssogelos, A. (2025). Make the West Great Again? Ukraine, Israel and the appropriation of the West by European populists. *European View*, 17816858251330420. refer 2
  - Shroufi, O. (n.d.). A critical discourse analysis of Geert Wilders’ pro-Israelism. *Critical Discourse Studies*, *0*(0), 1–18. refer 3
  - Brubaker, R. (2017). Between nationalism and civilizationism: The European populist moment in comparative perspective. *Ethnic and Racial Studies*, *40*(8), 1191–1226. refer 4
- Finally, a minor comment from the point of view of readability. In the section **‘Positions on ‘Europe’ and the Populism-Euroscepticism nexus’, I would suggest a clearer distinction between LPF and PVV: currently, the discussion of both **parties is in the same paragraph.

Overall, the report is practically ready. Addressing my remarks will just strengthen its argumentation.

 I wish the author all the best.

Is the case presented with sufficient detail to be useful for teaching or other practitioners?

Yes

Is the work clearly and accurately presented and does it cite the current literature?

Yes

If applicable, is the statistical analysis and its interpretation appropriate?

Not applicable

Are all the source data underlying the results available to ensure full reproducibility?

No source data required

Are the conclusions drawn adequately supported by the results?

Yes

Is the background of the case’s history and progression described in sufficient detail?

Partly

Reviewer Expertise:

political science; comparative politics; political communication; party politics; discourse studies

I confirm that I have read this submission and believe that I have an appropriate level of expertise to confirm that it is of an acceptable scientific standard.

## References

[1] : The neoliberal Great Replacement theory: on the racialization of markets and migration. *Distinktion: Journal of Social Theory* .2026;27(1) : 10.1080/1600910X.2025.249982783-101 10.1080/1600910X.2025.2499827

[2] : Make the West Great Again? Ukraine, Israel and the appropriation of the West by European populists. *European View* .2025;24(1) : 10.1177/17816858251330420115-123 10.1177/17816858251330420

[3] : A critical discourse analysis of Geert Wilders’ pro-Israelism. *Critical Discourse Studies* .2025; 10.1080/17405904.2025.25742651-18 10.1080/17405904.2025.2574265

[4] : Between nationalism and civilizationism: the European populist moment in comparative perspective. *Ethnic and Racial Studies* .2017;40(8) : 10.1080/01419870.2017.12947001191-1226 10.1080/01419870.2017.1294700
